# Potential of CRISPR/Cas system as emerging tools in the detection of viral hepatitis infection

**DOI:** 10.1186/s12985-023-02048-5

**Published:** 2023-05-08

**Authors:** Howra Bahrulolum, Hossein Tarrahimofrad, Fatemeh Nouri Rouzbahani, Saghi Nooraei, Mehdi Mousavi Sameh, Abbas Hajizade, Gholamreza Ahmadian

**Affiliations:** 1 Department of Industrial and Environmental and Biotechnology, National Institute of Genetic Engineering and Biotechnology (NIGEB), Tehran, 1497716316 Iran; 2 Department of Animal Biotechnology, National Institute of Genetic Engineering and Biotechnology (NIGEB), Tehran, 1497716316 Iran; 3 Applied Microbiology Research Center, Systems Biology and Poisonings Institute, Baqiyatallah University of Medical Sciences, Tehran, 1435916471 Iran

**Keywords:** Viral hepatitis, CRISPR/Cas system, Virus diagnosis tools

## Abstract

Viral hepatitis, the most common cause of inflammatory liver disease, affects hundreds of millions of people worldwide. It is most commonly associated with one of the five nominal hepatitis viruses (hepatitis A–E viruses). HBV and HCV can cause acute infections and lifelong, persistent chronic infections, while HAV and HEV cause self-limiting acute infections. HAV and HEV are predominantly transmitted through the fecal-oral route, while diseases transmitted by the other forms are blood-borne diseases. Despite the success in the treatment of viral hepatitis and the development of HAV and HBV vaccines, there is still no accurate diagnosis at the genetic level for these diseases. Timely diagnosis of viral hepatitis is a prerequisite for efficient therapeutic intervention. Due to the specificity and sensitivity of clustered regularly interspaced short palindromic repeat (CRISPR)/CRISPR-associated sequences (Cas) technology, it has the potential to meet critical needs in the field of diagnosis of viral diseases and can be used in versatile point-of-care (POC) diagnostic applications to detect viruses with both DNA and RNA genomes. In this review, we discuss recent advances in CRISPR–Cas diagnostics tools and assess their potential and prospects in rapid and effective strategies for the diagnosis and control of viral hepatitis infection.

## Background

Viral hepatitis, an infection that causes inflammation of the liver, is a significant global health-care problem. There are numerous viruses known to cause liver inflammation, including Herpes simplex virus (HSV), Cytomegalovirus (CMV), Epstein–Barr virus (EBV), and varicella-zoster virus (VZV) [1]. However, the most frequent causative agents of this condition are the hepatotropic viruses, including Hepatitis A virus (HAV), Hepatitis B virus (HBV), Hepatitis C virus (HCV), Hepatitis delta virus (HDV), and Hepatitis E virus (HEV), which lead to either an acute or a chronic infection [2]. These liver viruses include a variety of DNA and RNA viruses from different viral families that are spread through different transmission routes [3]. Globally, viral hepatitis causes about 1.4 million deaths each year. In terms of mortality, HBV and HCV rank among the world’s top four infectious threats, on par with HIV, malaria, and tuberculosis [4, 5]. The clinical symptoms of viral hepatitis varies from symptoms such as asymptomatic hepatitis to acute hepatitis, acute liver failure, chronic liver disease and even the development of liver-related outcomes including hepatocellular carcinoma (HCC) in different patients [6]. Despite some medical advances in hepatitis therapy and the development of HAV and HBV vaccines, there is still a large gap in the accurate genetic diagnosis of viral hepatitis infections. Early and accurate diagnosis of viral hepatitis and early evaluation of its prognosis is critical for the effective treatment and care of affected people [7]. The emerging gene editing technology, CRISPR/Cas, has the potential to fill these technical gaps. The CRISPR/Cas system is attractive for a wide range of diagnostic and therapeutic applications with high efficiency and programmable designs [8]. CRISPR loci were first discovered in *E. coli* in 1987, followed by the identification of CRISPR-dependent proteins. Subsequent research has shown that CRISPR serves as a defense mechanism in prokaryotic cells against exogenous genetic elements, including viral pathogens and plasmids, by precise and specific degradation of their sequences [9, 10]. The CRISPR-Cas system is divided into two main categories, Class I and II, which include six types and forty-eight subtypes [11]. Class I includes types I, III, and IV, which are associated with the enzymes Cas3, Cas10, and Cas8-like (csf1), respectively, and use CRISPR RNA (crRNA) along with a set of Cas proteins to identify and eliminate the target genetic elements. Class II of the CRISPR system includes types II, V, and VI, which contain Cas9, Cas12, and Cas13 proteins, respectively, and uses crRNA and only one multi-domain Cas protein for its function [12–16]. In this review, we explore the recent advances in CRISPR–Cas diagnostic tools and their potential applications in the detection of viral hepatitis infections. Firstly, we overview in brief the five main classifications of hepatitis viruses and their clinical aspects. In the following sections, we outline the importance and applications of the CRISPR-Cas system for infectious disease diagnostics, mainly focusing on different CRISPR-Cas effectors for the detection of viral hepatitis. We will also provide a detailed explanation of the pros and cons of CRISPR-based diagnostic systems and introduce future research perspectives.

## Viral Hepatitis

Viral hepatitis is one of the most common causes of human morbidity and mortality, both due to acute infection and chronic complication [17, 18]. Although all five hepatitis viruses mainly infect the liver, they are classified into different viral families and are completely different from each other in terms of structure, genome and life cycle [19]. HAV and HEV are usually transmitted from person to person through the fecal-oral route, while HBV, HCV, and HDV are transmitted through exposure to the blood and body fluids of an infected person [19]. Viral hepatitis disease often occurs without any symptoms. However, chronic hepatitis can evolve into jaundice, anorexia, fibrosis, and eventually cirrhosis and liver cancer. Individuals with hepatitis initially experience flu-like symptoms, similar to many other acute viral infections. More possible symptoms include fatigue, fever, joint and muscle pain, jaundice (yellowing of the eyes and skin) as well as abdominal pain. In most cases, the host’s immune response is able to viral elimination. However, this rate varies from 100% clearance in cases of HAV to only 20–30% clearance in acute cases of HCV. When HBV and HCV infections persist for up to 6 months, they can be defined as chronic hepatitis B and C, respectively. Each viral hepatitis is diagnosed by a patient history, physical examination, liver function tests, antibody serology tests, and a polymerase chain reaction (PCR)-based test for viral nucleic acid (RNA or DNA). Humans are the only known hosts for the hepatitis viruses, except for HEV, which may have a reservoir in domestic animals [20].

### Hepatitis A

HAV was first identified in 1973. It is a non-enveloped, icosahedral, single-stranded RNA virus belonging to the picornavirus family. At present, HAV can be divided into three genotypes including I, II, III, with two subtypes including subtypes A and B, which can infect humans. It is usually transmitted through the fecal-oral route, either by person-to-person contact and eating or drinking contaminated food or water. HAV is endemic in many countries, especially countries with limited health care resources [21]. The prevalence of HAV infection is low in developed countries. The seroprevalence of anti-HAV antibodies in the United States is approximately 10% in children and 37% in adults. More than 50% of cases in developed countries are the result of infection from travel to endemic countries [22]. HAV can cause an acute infection that is usually self-limiting and does not lead to chronic infection or chronic liver disease. The incubation period for the HAV is between 15 and 50 days (28 days) from exposure. Individuals with underlying chronic diseases are at higher risk of developing acute liver failure due to HAV infection. The case-fatality rates estimate from 0.3 to 0.6% for all ages and it increases to 1.8% in patients over 49 years old [23]. The spread of HAV in the liver occurs through the portal vein (the blood vessel that brings blood to the liver from the intestines) after the virus passes through the mucosa of the small intestine wall. The viruses subsequently replicate in hepatocytes before excretion, again via bile through feces [24]. Acute hepatitis A is mainly diagnosed by the presence of immunoglobulin M antibody to HAV (IgM anti-HAV). Anti-HAV IgM becomes detectable a few days before or concurrently with the onset of symptoms. Serum IgM levels increase during acute infection and remain positive for an average of 4 months after the onset of symptoms. IgG anti-HAV appears early in the course of infection and is detectable for many years, conferring lifelong immunity. Although Nucleic acid amplification techniques (NAAT; such as PCR) are more sensitive than the immunoassay test for the detection of HAV, they are rarely used for this purpose. Sequencing and phylogenetic analysis are mainly used to trace outbreaks, and these techniques are particularly useful for identifying transmission routes [24, 25]. Currently available vaccines for HAV are safe and highly effective and are included for routine childhood immunization programs in different countries [26]. The standard vaccination schedule for the inactivated vaccine against HAV consists of two doses administered at least 6 months apart. This vaccination schedule has been shown to be highly protective and is expected to last for many years [27].

### Hepatitis B

HBV is a prototype member of the Hepadnaviridae family. It is an enveloped viruses which contains relaxed, circular, partial double stranded DNA [28]. Although HBV is a DNA virus, replication occurs through reverse transcription from pre-genomic RNA (pgRNA) [29]. Currently, HBV is classified into 10 genotypes, including genotypes A to J, based on differences in the genome sequence, with 35 sub-genotypes. The distribution of genotypes varies widely around the world [30]. HBV is one of the common causes of liver diseases including liver cancer and is highly contagious; it is transmitted through exposure to infected blood and other body fluids (particularly amniotic fluid, semen, and vaginal fluids) [31, 32]. It is 100 times more infectious than HIV and 10 times more than HCV, as well [22]. The prevalence of HBV infection varies in different regions of the world, ranging from 0.7% of the adult population in low-endemic areas such as North America, Western Europe, and parts of South America to 6.2% in high-endemic areas such as Africa, Southeast Asia, and China [30]. The overall prevalence of chronic HBV in the USA was about 3/5% [33]. HBV infection can lead to both acute (short-term) and chronic (long-term) disease. Acute HBV infection can be asymptomatic or symptomatic. Most infected adults recover, even if their signs and symptoms are severe, but 5–10% of infected adults develop chronic infection, potentially leading to cirrhosis and liver cancer [34]. HBV has a specific tropism for liver cells to which it adheres and fuses during primary infection. HBV is internalized into hepatocytes by endocytosis after binding to its high-affinity receptor, sodium taurocholate co-transporting polypeptide (NTCP). The capsid of the virus then released into the cytoplasm, and translocated to the nuclear pore via the microtubule network. At the nuclear pore, rcDNA is released in the nucleus where it is converted into covalently closed circular DNA (cccDNA). In this step cccDNA becomes chromatinized, and serves as the transcriptional template for all HBV pregenomic RNAs. In the cytoplasm, pgRNA is encapsidated and retrotranscribed into rcDNA. The capsids are either enveloped and secreted as new virions from the cell, or transported back to the nucleus to amplify the pool of cccDNA [35]. HBV DNA encoded several different proteins play an essential role in viral persistence and liver pathogenesis, including two core proteins, a particulate core antigen (HBcAg), and a secreted antigen (HBeAg), surface antigen (HBsAg), HBx and polymerase and all of which play a role in its diagnosis and surveillance [24, 36]. After infection, HBV DNA and HBsAg can be detectable in an infected person’s serum within 1 to 2 weeks. Six to eight weeks later, IgM anti-HBc and HBeAg become detectable. In acute HBV infection cases, HBsAg and HBeAg disappear within 4–6 months. The disappearance of HBsAg is followed by the appearance of Antibody to HBsAg (Anti-HBs). The interval between the disappearance of HBsAg from the serum and the appearance of anti-HBs in it is known as the window phase and may last up to 6 months [22]. Hence, the initial step of HBV diagnosis is performed using a serological test to detect HBsAg and other HBV antigens and antibodies. Further molecular tests for quantitative and qualitative detection are used to verify the first step of diagnosis (Table 1) [37]. Reactivation of HBV refers to a sudden increase in HBV replication in a patient with chronic or previous HBV. Although, HBV reactivation (HBVr) can occur spontaneously, it is more commonly induced by the immunosuppressive drug therapy (ISDT) for cancer, immunologic diseases, or transplantation [38]. Vaccination is a highly effective strategy for HBV infection prevention. A three-dose series of vaccination of Hepatitis B vaccines (Recombinant), composed of HBsAg, results in a protective level of anti-HBs titers in > 90% of healthy individuals [39].

**Table 1:** Serological marker of HBV infections

Serological marker	Significant of positive test
HBsAg	Present in acute or chronic infection. Indicates the patient is infectious
HBeAg	Present in acute or chronic infection. Indicates ongoing viral replication
Anti-HBs	Present after successful recovery from hepatitis B infection or/and after successful vaccination. Indicates immunity
Anti-HBc	Appears shortly after HBsAg in acute infection. Indicates previous or current HBV infection

### Hepatitis C

HCV, a member of the Flaviviridae family, has a positive-sense single-stranded RNA genome coding three structural and seven non-structural proteins. The high mutation rate of HCV leads to marked genomic heterogeneity. HCV is classified into seven confirmed genotypes and at least 67 confirmed subtypes. Meanwhile, genotypes 1–3 are globally distributed, while genotypes 4–6 are concentrated in a certain area. HCV genotype 4 and subtype 5a are endemic in the Middle East and northern parts of South Africa, respectively. HCV genotypes 6 and 7 are mainly observed in Southeast Asia and the Democratic Republic of Congo, respectively [24, 40]. The main forms of HCV transmission are through the blood borne route, such as intravenous and intranasal drug use, contamination of medicine injections, and by sexual contact [41]. The worldwide prevalence of HCV is estimated to be 1.5–2.3% in the most affected areas and 0.5–1.0% in other areas [42]. The prevalence of HCV in injection drug users is between 50 and 90%, which is the largest group among infected people [43]. HCV can cause acute infections with a high tendency for chronic disease. Chronic HCV can lead to severe liver disease including cirrhosis and the risk of HCC [44]. Scavenger receptor class B type I (SCARB1), Occluding (OCLN), Claudin-I (CLDN1) and Cluster of differentiation 81 (CD81) are the main host factors that facilitate the attachment and uptake of HCV into hepatocytes. Furthermore, CD81 interacts with the viral particle through the viral envelope glycoprotein E2 or other molecules, facilitating the entry of the viral particle into the hepatocyte [45]. Virological diagnosis of HCV is established by two categories of laboratory tests, including indirect tests, in which serological assays are performed to detect a specific antibody of HCV (anti-HCV) in the infected patient, and direct tests, in which the detection, quantifying, or characterization of the components of HCV viral particles such as HCV RNA and core antigen can be conducted. The sensitivity and specificity of third generation enzyme immunoassays (EIAs) for detecting antibodies against different antigens of the HCV viral particle is 99% after 2 to 6 months following exposure to the virus. One of the direct assay methods is the use of real-time PCR for the quantitative or qualitative detection of HCV RNA, which can detect HCV particles in patients with at last 10–15 IU/mL (Table 2) [46].

**Table 2:** Diagnostic tests for cleared, acute and chronic HCV.

Diagnosis Test	Prior/Cleared HCV Infection	Acute HCV Infection	Chronic HCV Infection
Antibody test/Anti-HCV (Indirect test)	Positive	Negative; becomes positive within 6–24 weeks	Positive
Viral load test/HCV RNA (Direct test)	Undetectable	Detectable within 1–2 weeks, usually very high load	Detectable

### Hepatitis D

HDV, a member of the genus *Deltavirus*, is an enveloped, circular, single-stranded negative-sense RNA virus that encodes two kinds of proteins; the small protein (S-HDAg) and the large protein (L-HDAg), which are 24 kDa and 27 kDa in size, respectively. S-HDAg is required for viral RNA replication, while the L-HDAg is essential for viral assembly [47]. HDV is considered a satellite virus because it requires the assistance of HBV surface proteins (HBsAg) to generate mature virion particles. As a result, two distinct patterns of infection for HDV can establish; either HBV/HDV coinfection or HDV superinfection. HBV/HDV coinfection occurs when a person simultaneously becomes infected with both HBV and HDV, whereas HDV superinfection occurs when a person who is already chronically infected with HBV acquires HDV. Development of a chronic HDV superinfection can exacerbate hepatic injury caused by HBV, and both coinfection and superinfection have been shown to result in more severe outcomes than HBV infection alone, including fulminant hepatitis, HCC, and chronic hepatitis [48]. Currently, three genotypes of HDV have been identified (Genotype I–III), among them Genotype I is predominant [22]. Like HBV and HCV, HDV is primarily transmitted sexually, as well as through exposure to infected blood and blood products [28]. Globally, it is estimated that about 5–10% of patients with chronic hepatitis B (CHB) are co-infected with HDV, with a higher prevalence in injecting drug populations. Areas with a high prevalence of HDV infection include the Mediterranean basin, South America, Mongolia, and Sudan. In North America and Northern Europe, the prevalence is low and mostly confined to injecting drug users [22, 49]. The first step in the diagnosis of HDV infection is testing HBsAg-positive individuals for the HDV-specific total antibodies (combined IgG and IgM) in the serum. For anti-HDV- antibodies positive patients the next step is testing for HDV RNA in serum to determine whether the antibody represents an ongoing active HDV infection (HDV-RNA-positive) or only represents a serologic scar (HDV-RNA-negative) [50].

### Hepatitis E

HEV was first discovered in 1983 when scientists were looking for the cause of the outbreak of non-A, non-B (NANB) hepatitis, which is transmitted enterically [51]. HEV belongs to the *Hepevirus* genus of the Hepeviridae family. [52, 53]. Three groups of mammalians Hepeviridae have been recognized based on full genome sequencing from human and animal strains. The first category consists of viruses that affect people, pigs, deer, and rabbits. The 24 sub-genotypes of the four human HEV genotypes (genotypes 1–4) identified from infected patients are included in this group [53]. HEV is most often transmitted through contamination of water and food, and less commonly through the zoonotic route or through blood transfusion [52]. HEV genotypes 1–4 show a selected geographical distribution. Genotypes 1 and 2 are the main causes of acute HEV infections in endemic areas in South Asia and Sub-Saharan Africa [54]. Genotype 3 is the most common genotype associated with HEV infection in Europe and America [55]. HEV genotype 4 was initially identified only in Asia, but further reports have also identified this genotype in pigs and humans in Europe [52]. HEV genotypes 1 and 2 are obligate human pathogens and are mainly transmitted through contaminated drinking water in areas with poor sanitation, while genotypes 3 and 4 are zoonotic and mainly transmitted through the consumption of raw or undercooked meat or liver products from its main host (i.e., pig, wild boars and deer) [56]. HEV infection is generally self-limited, and causes acute hepatitis. Infection with HEV has an incubation period of 15 to 60 days. Typical acute HEV may lead to elevation in aminotransferases to greater than 10 times than normal. The main clinical symptoms described are anorexia, nausea, malaise, and abdominal pain, in addition to arthralgias, diarrhea, pruritus, and a rash. Within 1 to 6 weeks after the infections, aminotransferases return to normal. The case fatality rate is approximately 0.5 to 3% in adult individuals. HEV can also cause fulminant hepatic failure (FHF), in which encephalopathy within days or weeks of the onset of symptoms develop. FHF appears to occur more commonly in pregnant women, hence HEV has a higher case fatality in pregnant women [22]. Both serological tests and nucleic acid-based tests have been developed for the diagnosis of HEV for epidemiological and diagnostic purposes. Serological assay for HEV infection usually depends on the detection of anti-HEV IgG and anti-HEV IgM. Anti-HEV IgM antibodies are usually detectable in the acute phase of the disease and its presence in serum lasts for approximately 4 or 5 months, indicating recent exposure, while anti-HEV IgG antibodies can persist for more than 10 years, indicating prior exposure. Therefore, the diagnosis of acute HEV infection is based on the presence of anti-HEV IgM, while the anti-HEV IgG diagnostic test is a good option for epidemiological investigations [57]. However, detection and quantification of HEV-RNA by PCR is a gold standard approach for the diagnosis of acute and chronic HEV infection [58]. Currently, no specific vaccines, except the one licensed in China, or therapeutic options are available for HEV [22].

## Different molecular methods for the diagnosis of viral infection

As infectious agents of potentially all types of living cells, viruses are the most abundant biological entities in the environment, that can cause an epidemic which threaten the life and health of humans and animals and cause significant economic loss. The occurrence of a serious outbreak often leads to irreversible losses or damage, so an early and rapid dignosis of the viral infectious is particulary important [59]. The traditional techniques for laboratory diagnosis of viral infections can usually divided into 3 categories including (1) direct detection of virions in patient material, viral antigens, or viral nucleic acids, (2) Virus isolation in cultured cells, followed by identification of the isolate (Indirect exam), and (3) serological diagnosis which are based on detection and measurement of antibodies in the patient’s serum [60]. The best viral diagnostics tool should satisfy the the following requirements criteria: speed, simplicity, sensitivity, specificity, and cost. Nucleic acid amplification test (NAAT), including PCR, real-time PCR, RT-PCR, nucleic acid sequence-based amplification (NASBA), and loop-mediated isothermal amplification (LAMP), and antibody-antigen complex detection-based methods such a**s** solid-phase enzyme immunoassays (EIAs) and enzyme-linked immunosorbent assay (ELISA) technology, in particular, have revolutionized diagnostic virology and are now widely used for detecting different viruses [61, 62]. Currently, NAATs are described as a ‘Gold Standard’ method for diagnosis of viral infections because of its high sensitivity and specificity. However, viral dignosis techniques based on PCR are limited by the high cost of reagents and instruments, as well as the harsh technical operation. The new generation analytical method based on the nonspecific DNA and RNA cleavage observed in CRISPR-Cas systems provide promising advances in CRISPR-based diagnostics of emerging infectious because of it’s high specificity, versatility, and rapid detection cycle [62, 63].

## CRISPR-based diagnostics systems: overview, and applications

The key discoveries that prokaryotic cells like bacteria and archaea have heritable adaptive immunity against foreign genetic elements mediated via CRISPR and CRISPR-associated (*Cas*) proteins has led to transformative advances in molecular biology [64]. Prokaryotic cells store these genetic elements from infectious agents such as bacteriophages, plasmids, or transposons in genomic locus called CRISPR arrays, allowing the cell to remember, recognize and clear infections. *cas* proteins facilitate adaptive immunity through the multistep process which comprises adaptation, CRISPR RNA (*crRNA*) biogenesis, and interference. During adaptation which is the first stage of CRISPR immunity, foreign genetic elements are recognized, processed and selected for integration into the CRISPR array, providing a recall element during recurrent infection. During the expression stage, also known as *crRNA* biogenesis, the CRISPR array is transcribed into a long precursor (pre-crRNA), and processed into mature form as crRNA. In the last stage, the mature crRNAs guide Cas proteins to cleave complementary sequences of foreign DNA (interference) and eliminate those elements. By uncovering the structural and functional components of these diverse systems, new tools, including those applicable to molecular diagnostics, are emerging [65].

### CRISPR-Cas: classification and mechanism of action

**Fig. 1:**
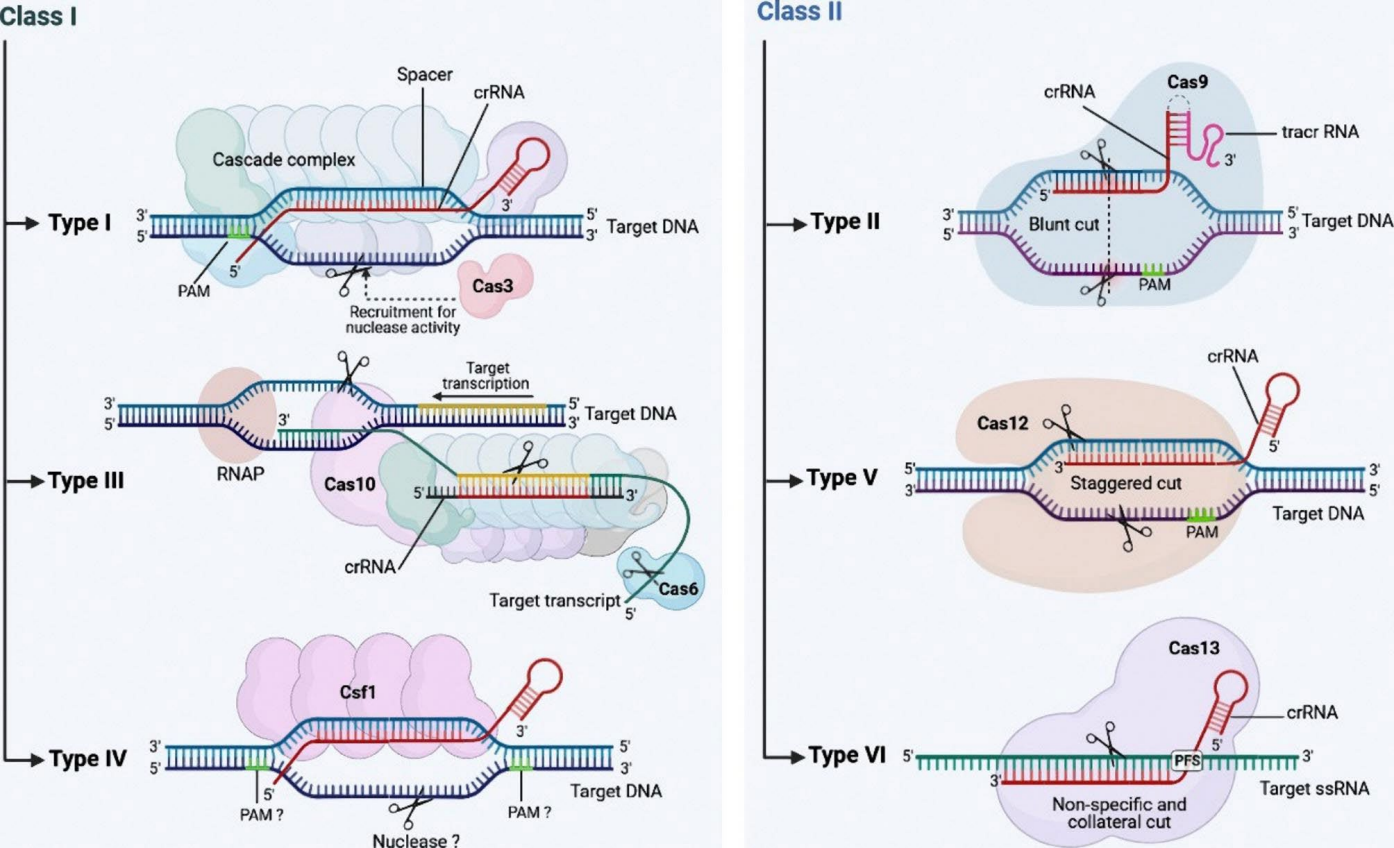
Classification of CRISPR-Cas system classes based on their effector proteins

Since the initial discovery of the CRISPR–Cas systems, its various variants have expanded rapidly. Currently, CRISPR-Cas systems are divided into two classes, six types and several subtypes based on evolutionary relationships. Depending upon the nature of the effector ribonucleoprotein complexes, two main classes of CRISPR-Cas systems have been defined; Class 1 (includes Types I, III, and IV) and Class 2 (includes types II, V, and VI). Class 1 systems are characterized by a complex of multiple effector proteins, and class 2 systems encompass a single crRNA-binding protein [11]. Most of the identified CRISPR-Cas systems (about 90%) belong to class 1 systems [16]. Among the diverse CRISPR systems, class 2 systems have primarily been applied for diagnostics, as these systems are simpler to reconstitute. They include enzymes with collateral activity, which serves as the backbone of many CRISPR-based diagnostic assays. Cas9, Cas12 and Cas13 have a major impact on the CRISPR-Cas classification for type II, type V and type VI as unique signature effector nucleases, respectively. The latest classification of CRISPR-Cas systems attributes the largest number of subgroups to the type V system with 17 derivative subgroups [11]. Class 1 systems (such as the type III effector nuclease Csm6 or Cas10) have also been engineered for diagnostics, either in combination with components of the class 2 system or with the native type III complex) [66]. A RuvC-like nuclease domain in the Cas12 system catalyzes the cleavage of a dsDNA target by Type V systems [67]. This feature has led to the use of Cas12a, also known as Cpf1, in viral pathogen diagnosis tests [68, 69]. The CRISPR-Cas type VI system has five subtype variants. Cas13a in subtype VI-A is known as an RNA-guided Rnase [16]. Cas13a is able to bind to crRNA and thus form a complex that eventually cleaves ssRNA [12]. The ability of Cas13a in ssRNA cleaves has led to the development of modern platforms for the rapid and sensitive detection of infectious diseases. Each of the Cas12a and Cas13a-based viral infection diagnosis platforms is discussed in the next section (Fig. 1) [70, 71].

### Applications of CRISPR-based diagnostics systems

Owing to the programmable nature of CRISPR-Cas systems, they have been exploited in many different fields of biology and medicine and, in fact, have revolutionized these fields. An increasing number of studies have shown that CRISPR/Cas technology can be integrated with biosensors and bioassays for molecular diagnostics. CRISPR-based diagnostics has attracted much attention as highly specific and sensitive sensors with easily programmable and device-independent capabilities. This technique has been used for the sensing of nucleic-acid-based biomarkers of infectious and non-infectious diseases and for the detection of mutations and deletions indicative of genetic diseases. With further research, it holds promise for detecting other biomarkers such as small molecules and proteins [66, 72]. In this section we briefly describe the application of CRISPR/Cas systems in the field of diagnostics.

#### CRISPR-cas in diagnostics of infectious diseases

The main goal of CRISPR-based diagnostics approaches are the identification of specific pathogens, such as the DNA or RNA viruses, bacteria and parasites. RNA viruses which detected with CRISPR-based approach include parvovirus B19, Zika virus (ZIKV), Dengue virus (DENV), Japanese encephalitis virus, Ebola virus (EBOV) and Corona virus. Besides RNA viruses, DNA viruses such as cytomegalovirus (CMV), Epstein–Barr virus, BK virus (BKV) and human papillomavirus (HPV) have also been sensed with CRISPR-based diagnostics [73]. CRISPR/Cas-system have also been utilized for the detection of various pathogenic bacteria like *Mycobacterium tuberculosis*, *Staphylococcus aureus*, *Listeria monocytogenes*, *Pseudomonas aeruginosa* and *Salmonella enteritidis* [74]. Moreover, the technique of CRISPR/Cas has been successfully applied for ultrasensitive detection of *Plasmodium parasite* responsible for malaria [75].

#### CRISPR-cas in diagnostics of non-infectious diseases

CRISPR-Cas-based diagnostics technologies have also been implemented for the detection of genetic material relevant to non-infectious diseases. For example, this system was used to sense abnormal levels of human CXCL9 mRNA, an indicator of acute cellular kidney-transplant rejection, in order to detection of graft-versus-host disease in kidney transplants [76]. Apart from mRNAs, CRISPR-based diagnostics system has also been used to detect specific miRNAs such as miR-19b in medulloblastoma patients’ serum [77] and miR-17 isolated from breast cancer cell lines [78].

#### CRISPR-cas in diagnostics of SNPs and small deletions

A key strength of CRISPR-based diagnostics is the single-nucleotide specificity of Cas enzymes, which permits the discrimination of point mutations (SNPs) and small deletions. Single-nucleotide specificity of CRISPR-based detection has made possible this system to sense markers of antimicrobial resistance, deletions and mutations in the epidermal-growth-factor-receptor gene *(EGFR*) and BRAF [79], mutations conferring Duchenne muscular dystrophy [80] and SNPs in the E3 ubiquitin ligase gene conferring eye color. The specificity potential of the CRISPR–Cas system has also been leveraged for the detecting of miRNAs, which are challenging to diagnose because of their short size and because they can differ by only a single base [66].

### ***In vitro*** viral infections detection based on the CRISPR/Cas system

Accurate, sensitive, and rapid diagnosis of viral infections is the critical first step toward accurate and timely treatment of viral infections. CRISPR-Cas-based diagnosis of pathogens has gained tremendous popularity over the past few years, mainly due to the high specificity, sensitivity, and rapid diagnosis of this system [81]. Numerous variations and advancements have been introduced to the CRISPR-based platform, counting on the collateral activity of CRISPR- Cas type V and type VI proteins, but the general conception remains unchanged. In the following, we outlined the various platforms based on the CRISPR-associated nucleases Cas9, Cas12, or Cas13 using for diagnosis of different viral infections.

#### CRISPR/Cas9 system–mediated diagnosis

In 2016, Pardee et al. introduced the first CRISPR/Cas-based diagnostic method able to recognize dsDNA. They developed a rapid and inexpensive diagnostic method for detecting Zika virus using CRISPR type II, called nucleic acid sequence-based amplification-CRISPR cleavage (NASBACC). The NASBACC system consists of three parts: nucleic acid sequence-based amplification for the isothermal preamplification of targets, protospacer adjacent motif (PAM)-dependent target DNA recognition by the Cas9, and a toehold detector for the readout. Briefly, a toehold switches triggered binds to a NASBA-amplified RNA fragment via reverse transcription. If the PAM sequence is available in the RNA fragment, Cas9-mediated cleavage causes a short RNA without the trigger sequence. In the absence of the PAM sequence, the trigger containing the full-length RNA activates the toehold switch, indicated by a color change [82]. However, in the same year when the collateral cleavage activity of Cas13a was discovered, this field was revolutionized. The collateral activity demands cleavage of non-targeted single-stranded RNA (ssRNA; Cas13) and single-stranded DNA (ssDNA; Cas12) in a solution, enabling the sensing of nucleic acids through signal amplification and empowering multiple readouts through the addition of functionalized reporter nucleic acids. In recent studies, various CRISPR-based methods, such as DNA Endonuclease-Targeted CRISPR Trans Reporter (DETECTR) and Specific High-Sensitivity Enzymatic Reporter UnLOCKing (SHERLOCK) techniques, have been developed that use type V CRISPR-CAS12a and Type VI Cas13a enzymes, respectively. The SHERLOCK system uses Cas13 endonuclease to degrade RNA strands and the DETECTR system uses the Cas12a protein to do the same on DNA [16, 68].

#### CRISPR/Cas13 system–mediated diagnosis

**Fig. 2:**
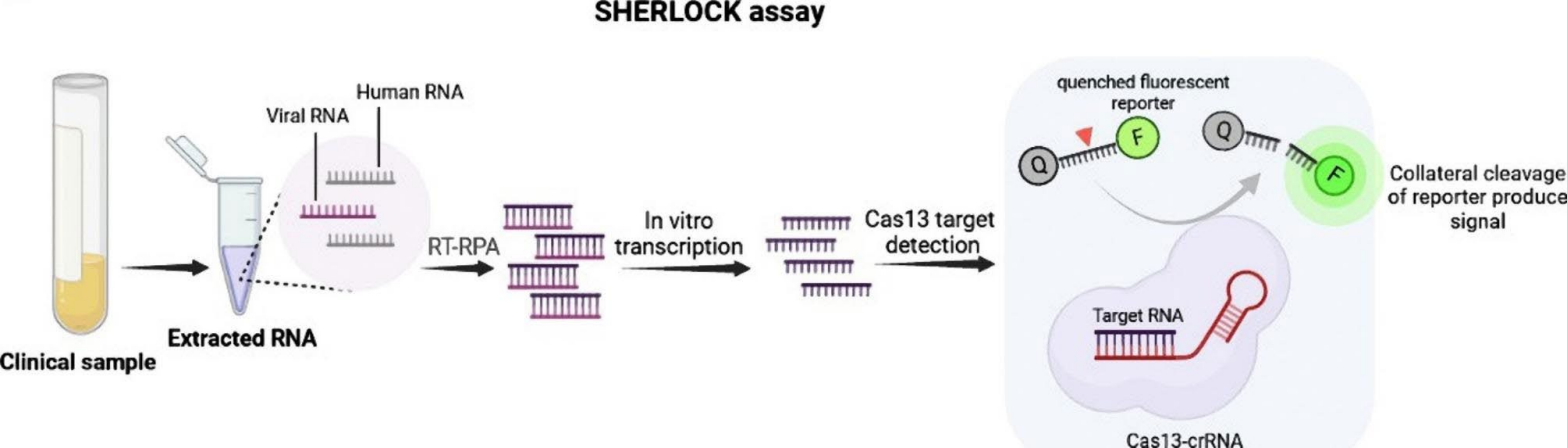
SHERLOCK methods in nucleic acid detection based on CRISPR technology

The first applicable platform based on the CRISPR–Cas type VI (Cas13) system, SHERLOCK, was developed in 2017 by the Zhang Lab based on the activity of Cas13 nuclease from Leptotrichia wadei [16]. SHERLOCK CRISPR–Cas system refers to novel nucleic acid detection using Cas13 or Cas12 nucleases paired with an isothermal pre-amplification step to increase the amount of DNA or RNA exhibiting high levels of collateral RNase activity upon the recognition of a specific target sequence. The SHERLOCK system consists of three basic steps: (1) The first step is isothermal preamplification, which generates multiple copies of an RNA or DNA template using specific primers. (2) Next, the forward primer containing a T7 promoter is integrated into the amplicons during the RPA reaction. T7 promoter allows T7 RNA transcription of dsDNA to convert it to ssRNA amplicons to provide targets for the Cas13a from the Leptotrichia wadeii (LwaCas13-crRNA) complex. Recognition and base pairing between crRNA and the target sequence activates LwaCas13a collateral activity, leading to sequence-unspecific degradation of adjacent quenched-RNA reporters and (3) in the last step, fluorescence-based nucleic acid detection with LwaCas13a is accomplished by incorporating a fluorescence probe that emits a detectable signal. ssRNA reporter molecules are composed of a fluorophore and a quencher, which are connected to each other by a short RNA oligomer, which, after separation, allows the separation of the fluorophore from the quencher and, as a result, emits a fluorescence signal that determines the presence of the RNA viruses (Fig. 2). The SHERLOCK system is highly sensitive and is able to detect one attomolar (aM or 10–18 M) of the target [66, 83].

Recently, a second version of the SHERLOCK system called SHERLOCKv2 has been developed, which takes about 2 h to complete and also increases the detection sensitivity to two attomolar [79]. In this platform, Cas protein is added to the sample along with the crRNA (designed to specifically bind to the target sequence) and ssDNA probes (as reporters). The probes are linked to a fluorophore at one end and to a quencher at the other end, and if there is a target sequence in the sample, the crRNA binds to that sequence and the collateral activity of Cas13a will be activated and cleaves the probes. As a result, fluorophores are released and a stable and strong fluorescent signal is detected by a fluorimeter [84]. The superiority of SHERLOCKv2 over SHERLOCK is important in that the entire SHERLOCKv2 reaction is performed in one step by adding the sample directly to the test strip. In addition, no purification and separation of nucleic acids is required on the samples [85]. Various platforms based on the Cas13 protein have evolved. Myhrvold et al. developed HUDSON (heating unextracted diagnostic samples to obliterate nucleases) and combined it with SHERLOCK. Through this combination, they were able to directly detect dengue and Zika viruses in patient body fluids at very low concentrations (1 copy per microliter). The advantage of this platform is that there is no need for pretreatment of the samples [86].

#### CRISPR/Cas12 system–mediated diagnosis

**Fig. 3:**
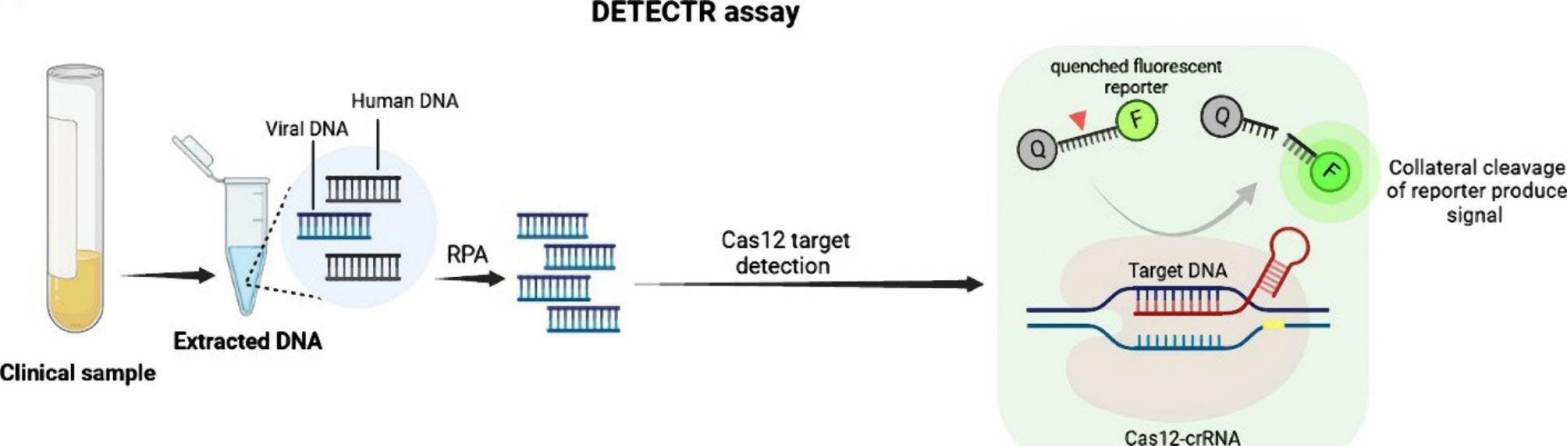
DETECTR methods in nucleic acid detection based on CRISPR technology

Cas12a is another Cas enzyme with collateral activity that detects DNA molecules. One of the first Cas12a-based detection platforms developed in 2018 by Doudna et al. was DETECTR [86]. In this system, Cas12a protein from Lachnospiraceae bacterium (LbCas12a) with non-specific collateral activity is guided to dsDNA targets by a complementary crRNA, triggering collateral cleavage of short ssDNA reporters carrying a fluorophore and a quencher. Similar to SHERLOCK, target recognition and reporter cleavage lead to the separation of the fluorophore from the quencher, which emits a fluorescence signal (Fig. 3) [87]. Cas12a has relatively weak collateral activity. Therefore, the Cas12-based diagnostic methods have low sensitivity for nucleic acids detection. With incorruption of isothermal preamplification by RPA, DETECTR reached attomolar sensitivity for the detection of DNA, which enables them to detect low target concentrations (2 aM concentrations) [88]. In other Cas12-based techniques, Li et al. used the collateral activity of this enzyme and developed a detection platform called HOLMES (a One-Hour Low-cost Multipurpose Highly Efficient System) for the rapid detection of DNA and RNA targets. The basis of HOLMES is similar to SHERLOCK in that in the presence of target DNA in the sample, the crRNA (in complex with Cas12a) binds to it, a ternary complex (CAS12a-crRNA-target DNA) is formed and the collateral activity of the Cas enzyme is turned on and the probes are degraded [87]. These are the most used CRISPR-based detection platforms for identifying pathogens; however, there are other platforms, such as Cas9- finding low abundance sequences by hybridization (FLASH) and Cas14-DETECTOR, that their explanation demands another review paper [89, 90].

Until now, many DNA and RNA viruses have been diagnosed using these methods. The CRISPR-Cas-based SHERLOCK system allows the detection of Zika virus, Ebola virus, and Dengue virus with a sensitivity of about 1 copy per microliter of sample. It has also enabled the detection of West Nile virus (WNV), Yellow fever virus (YFV), and severe acute respiratory syndrome coronavirus 2 (SARS-CoV-2). Similarly, DETECTR technology has also been employed to detect SARS-CoV-2. Furthermore, human papillomavirus (HPV) strains (HPV-16 and HPV-18) can be diagnosed by DETECTR technology. In the next section, the potential application of different CRISPR-Cas systems in the diagnosis of viral hepatitis are discussed [91].

## Potential of CRISPR/Cas system in viral hepatitis diagnosis

Achieving the World Health Organization’s goal of eliminating viral hepatitis by 2030 requires accurate diagnosis of the disease. As mentioned, the ideal diagnostic assay should provide accurate and sensitive identification of the viruses while being affordable, portable, and able to distinguish different variants. Developing new tools which meet the requirements of the WHO standard diagnostic test can completely reshape epidemiological surveillance and medical health care systems for the viral hepatitis in the world [85]. In this regard, the emerging CRISPR/Cas system can provide an opportunity as a promising precision diagnostic tool, especially for HBV DNA and HCV RNA, which are the most important indicators of viral hepatitis, by targeting viral DNA or RNA. In this section, we review the studies conducted in the field of hepatitis viruses’ sensing using different CRISPR-Cas platforms.

### A CRISPR-Cas–based platform for detection of HBV

Chen X et al. devised a CRISPR-Cas based detection platform, known as “CRISPRHBV”, for ultrasensitive and early detection of two major HBV genotypes (HBV-B and HBV-C) in clinical applications. CRISPR-HBV consists of three steps including multiple crossover displacement amplification (MCDA) for rapid pre-amplification, Cas12b-based detection to decode targets, and result readout with real-time fluorescence and lateral flow biosensors. This system is able to detect 10 copies of genomic DNA per test and has 100% specificity and also has no cross-reactivity in other HBV genotypes and pathogens. The entire assay process, which includes DNA template extraction, MCDA preamplification reaction, CRISPR-Cas12b-based detection, and readout of results can be completed in 60 min and does not require expensive equipment. Additionally, the feasibility of the CRISPR-HBV assay for HBV-B and -C genotyping was successfully confirmed by validation with clinical samples. Therefore, the CRISPR-HBV system has the potential to be a POC diagnostic test for the detection and diagnosis of HBV genotypes B and C in clinical applications, especially in countries with insufficient medical resources [92].

Since the formation of an intranuclear reservoir of cccDNA in the hepatocyte is the main cause of persistent HBV infection, Zhang, X et al. developed highly sensitive and specific diagnostic tools for the detection of HBV cccDNA based on CRISPR-Cas13a technology. This technique includes the following steps: (1) using plasmid-safe ATP-dependent DNase (PSAD) and HindIII enzymes to digest circular rcDNA and linear double-stranded DNA, (2) amplification of specific HBV cccDNA fragments using rolling circle amplification (RCA) and PCR, and (3) target detection using the CRISPR-Cas13a system. This assay can detect 1 copy/µL HBV cccDNA by CRISPR/Cas13-assisted fluorescence readout. Furthermore, by performing ddPCR, qPCR, RCA-qPCR, PCR-CRISPR and RCA-PCR-CRISPR, this assay was validated for the detection of cccDNA in HBV-associated liver tissues, plasma, whole blood, and peripheral blood mononuclear cells (PBMCs) [93].

Many studies revealed that drug-resistance mutations occurring in the HBV genome can increase the risk of HBV transmission or cause active replication of the viral genome, resulting in a lytic infection and other clinical problems in affected patients. One of the most important factors in antiviral drug resistance is the mutation in the YMDD motif of the HBV P gene. Therefore, Wang S. et al. developed a highly sensitive and practical method for the detection of HBV DNA and YMDD drug resistance mutation based on the CRISPR-Cas13a detection system and PCR amplification, and evaluated its diagnostic capability using clinical samples. This technique represents very high sensitivity for detecting HBV and identifying drug resistance mutations using conventional PCR. Given that PCR amplification is more stable than isothermal amplification techniques, PCR was used to target amplification prior to CRISPR-Cas13a detection. In terms of sensitivity, it was found that this technique can detect one copy per test for HBV DNA and YMDD drug resistance mutations [94]. In another study, Ding, R et al. developed a fast and sensitive HBV POC test based on LAMP-Coupled CRISPR-Cas12a. This assay creatively solves the problem of the POC technique within 10 min, especially the nucleic acid sample extraction problem. Based on Loop isothermal amplification (LAMP)-Cas12a, visualization of assay results is provided with both fluorescent and lateral flow test strips. High-sensitivity real-time detection can be achieved in fluorescence readout, while results visible to the naked eye can be achieved with lateral flow test strip technology. The fluorescent readout-based Cas12a assay can achieve HBV detection with a sensitivity of 1 copy/µL in 13 min, while the lateral flow test strip technique takes only 20 min. The evaluation of clinical samples shows the sensitivity and specificity of both the fluorescence readout method and the lateral flow test strip 100%. Additionally, the assay results were completely comparable with qPCR. The LAMP-Cas12a-based HBV technique relies on minimal equipment and low costs to provide rapid and accurate test results, thereby offering significant practical potential for POC HBV detection in medically underserved regions (Table 3) [95].

### A CRISPR-Cas–based platform for detection of HCV

The genetic diversity of HCV and quasispecies generated during replication have led to viral resistance to direct-acting antivirals (DAAs), as well as obstacles to the development of a vaccine. The accurate and timely diagnosis of disease is a prerequisite for efficient therapeutic intervention and epidemiological surveillance [96]. While techniques such as immunoassay and qPCR play an important role in the diagnosis of HCV, rapid and accurate POC testing is important to identify individuals with HCV, particularly in resource-limited settings where access or availability of molecular testing is still limited. Therefore, to fill this gap, there is a need to develop a new molecular assay for the rapid detection of HCV RNA in resource-limited settings [97]. Recently, Kham-Kjing et al. developed and evaluated a fast and sensitive method for the detection of HCV RNA. The method was based on a reverse transcription loop-mediated isothermal amplification (RT-LAMP)-coupled CRISPR–Cas12 system that allows the detection of specific HCV genome sequences. Amplified products after cleavage reactions can be visualized on lateral flow bands or measured with a fluorescence detector. When tested on clinical samples from individuals infected with HCV, HIV, or HBV, or from healthy donors, the CRISPR-Cas12 assay combined with RT-LAMP compared to the reference method, which was Roche COBAS AmpliPrep/COBAS TaqMan HCV Test, showed 96% sensitivity, 100% specificity and 97% agreement. This assay was able to detect HCV RNA concentrations as low as 10 ng/µL. Therefore, this accurate and specific technique has the potential to be a cost-effective and reliable POC test to identify individuals with HCV in low-resource settings (Table 3) [98].

**Table 3:** Applications of CRISPR/Cas system in viral hepatitis detection

Types of Hepatitis	Platform Name	Cas Protein	Amplification Methods	Visualization	Sensitivity	Times	References
HBV	CRISPRHBV	Cas12b	MCDA	Real-time/ Fluorescence	10 copies/µL	60 min	[91]
	-	Cas13a	RCA/PCR	Fluorescence	1 copy/µL	-	[92]
	PCR-CRISPR	Cas13a	PCR	Fluorescence	1 copy/µL	15 min	[93]
				Fluorescent		13 min	
	LAMP-Cas12a-based HBV	Cas12a	LAMP	Lateral flow test strips	1 copy/µL	20 min	[94]
HCV	RT-LAMP-Coupled CRISPR–Cas12	Cas12a	RT-LAMP	Fluorescent/ Lateral flow test strips	10 ng/µL	-	[97]

These overall studies proved the potential of the CRISPR/Cas system as an effective diagnostic tool for viral hepatitis infections. Beyond these studies describe above, till now, no CRISPR-based diagnosis method has been developed for the detection of HAV, HDV, and HEV. However, Cas12a enzyme is the enzyme of choice for developing a CRISPR-based method for HAV detection. As well as, since HDV is an RNA-virus, Cas13a is the choice enzyme for CRISPR-based diagnosis method for HDV. Like HDV, HEV is an RNA-virus, so Cas13a is the choice enzyme for CRISPR-based diagnosis method for the detection of this virus. Uncertainty, the potential of the CRISPR/Cas system can soon become the leading diagnostic tool for different viral hepatitis infection.

## Limitation and future perspectives of CRISPR/Cas-based diagnostics system

Within a few years, CRISPR-based molecular diagnostics system have been developed from an experimental tool for nucleic acid biosensing to a clinically viable diagnostic tests for the fast, affordable, sensitive, and specific detection of pathogens including viruses at the POC. Although this technology apparent many advantages, it has several challenges and limitations which needs to overcome for safe and efficient clinical application. The major limitations of most current CRISPR-Cas-based diagnostic tools including SHERLOCK and DETECTR is their dependence on preamplification of the target nucleic acids by either PCR or isothermal amplification processes (e.g., PCR, RPA, or LAMP). However, the isothermal amplification process often needs a set of proteins such as; polymerase, recombinase, or binding proteins and primers which includes 2 primers for RPA and 4 to 6 primers for LAMP and additional sample preparation steps, which greatly complicates and lengthen the assay time for 20 min to 2 h [99]. To resolve these problem, various amplification-free detection methods such as digital CRISPR, multiple crRNAs and highly sensitive signal transduction methods have been developed in recent years for direct detection of unamplified samples, which can achieve rapid, highly sensitive detection of viruses. However, these methods also require specific equipment like high-cost fluorescent or optical detection instruments, microfluidic chips, etc., which limits their wide application [100]. It can be predicted that the future CRISPR/Cas technology platform will still rely on the support of other technologies. For example, by combining more signal readout techniques with nanomaterials, simpler and more sensitive analytical performance for more diverse applications of the unique features of the CRISPR/Cas system will be achieved.

In addition, the ease of use of CRISPR-based diagnostics is improved through optimized one-pot reactions and simple visualization of test results. However, sample preparation still requires a separate step and incubation temperatures higher than room temperature require heating devices. Furthermore, target concentrations close to the lowest analyte concentration (LOD) of the assay make it challenging to quantify the readout with certainty, especially when using a lateral flow assay. For home or point-of-care (POC) use, assay design should combine simple sample preparation protocols with robust detection methods to provide robust results in variable or challenging condition, such as long-term storage, limited user training, and harsh environmental conditions. In order to achieve this goal, the integration of sample preparation, measurement, and reporting into single, easy-to-operate units are becoming feasible using microfluidic techniques [66]. Future studies should focus on developing a more user-friendly, one-step diagnostic approach involving viral nucleic acid release, pre-amplification, CRISPR-Cas mediated detection and signal readout.

Another disadvantage of CRISPR-Cas systems particularly Cas9 is the ‘off-target’ effect, in which Cas9 binds to unintended genomic sites for cleavage. Such off-target effect can potentially lead to false negative or positive results. This issue can be overcome with the use of bioinformatic software for designing an appropriate sgRNA [101].

Automation and artificial intelligence can greatly strengthen the application of CRISPR-Cas systems in viral diagnostics. CRISPR-Cas-based diagnostic systems can be combined with artificial intelligence (AI) to provide an early warning system for rapid, affordable, accurate, and smart detection of an infectious agent. In particular, user-friendly and portable CRISPR-Cas-based diagnosis kits are provided to hospitals, health centers, communities, or even individuals for the fast and accurate detection of specific pathogens. Smartphone retrieval of the test result is achieved through an application. Data from various locations can be stored and processed in a cloud computing system via the 5G service, which can be accessed by restricted personnel for support decisions or publicly for research purposes. The cloud computing system calculates the risk of infection to generate an AI-equipped model by updating diagnostic items, and then warn policymakers and individuals [102–104]. Such a CRISPR-Cas diagnostic AI-powered alarm system provides early warning about the risk of nearby infectious viruses and would be very useful for timely response and appropriate measures to prevent the spread of viral infections.

## Conclusions

Limitations and drawbacks in traditional diagnostic techniques open new ways to use sensitive and efficient molecular diagnostic methods for the rapid diagnosis of viral infections. Type II (Cas9), V (Cas12), and VI (Cas13) CRISPR-based systems provide advanced diagnostic tools for the rapid detection and control of DNA and RNA viruses. The development of new CRISPR-based platforms for the molecular diagnosis of viral hepatitis promises to change healthcare and improve the epidemiological management of this infectious disease globally. Currently, developed CRISPR-based assays must be validated by clinical trials and assay validation should be monitored and maintained after clinical implementation to ensure their performance. Despite many successful developments in the CRISPR-Cas-based diagnostic platform, there is still a long way to go from the laboratory to practical or clinical use.

## Data Availability

Not applicable.

## List of Abbreviations

HSV: Herpes simplex virus
CMV: Cytomegalovirus
EBV: Epstein–Barr virus
VZV: varicella-zoster virus
HAV: Hepatitis A virus
HBV: Hepatitis B virus
HCV: Hepatitis C virus
HDV: Hepatitis delta virus
HEV: Hepatitis E virus
HCC: Hepatocellular carcinoma
CRISPR-Cas: Clustered regularly interspaced short palindromic repeat-CRISPR-associated sequences
POC: Point-of-care
PCR: Polymerase chain reaction
NAAT: Nucleic acid amplification techniques
dsDNA: double-stranded DNA
cccDNA: covalently closed circular DNA
SCARB1: Scavenger receptor class B type I
OCLN: Occluding
CLDN1: Claudin-I
CD81: Cluster of differentiation 81
EIAs: Enzyme immunoassays
CHB: Chronic hepatitis B
FHF: Fulminant hepatic failure
EM: Electron microscopy
qPCR: quantitative PCR, NASBA:Nucleic acid sequence-based amplification
LAMP: Loop-mediated isothermal amplification
LCR: Ligase chain reaction
RT-PCR: Reverse transcriptase PCR
dPCR: Digital PCR
NGS: Next-generation sequencing
NASBACC: Nucleic acid sequence-based amplification-CRISPR cleavage
DETECTR: DNA Endonuclease-Targeted CRISPR Trans Reporter
SHERLOCK: Specific High-Sensitivity Enzymatic Reporter UnLOCKing
PAM: Protospacer adjacent motif
HOLMES: One-Hour Low-cost Multipurpose Highly Efficient System
FLASH: Finding low abundance sequences by hybridization
WNV: West Nile virus
YFV: Yellow fever virus
SARS-CoV-2: Severe acute respiratory syndrome coronavirus 2
HPV: Human papillomavirus
IFN-α: Interferon-α
NA: Nucleoside analogs
APOBEC3: Apolipoprotein B mRNA-editing catalytic polypeptide-like 3
rcDNA: Relaxed circular DNA
pgRNA: Pregenomic RNA
MCDA: Multiple crossover displacement amplification
PSAD: Plasmid-safe ATP-dependent DNase
RCA: Rolling circle amplification
PBMCs: Peripheral blood mononuclear cells
LAMP: Loop isothermal amplification
DAAs: Direct-acting antivirals
IRES: Internal ribosomal entry site.

## References

[1] Tapper EB, Curry MP. *Hepatitis Caused by Other Viruses* Handbook of Liver Disease, 2018: p. 78.

[2] Sarin SK, Kumar M. *Viral hepatitis A*, in *Molecular Pathology of Liver Diseases*. Springer; 2011. pp. 527–52.

[3] Qu B, Brown RJ (2021). Strategies to inhibit Hepatitis B Virus at the transcript level. Viruses.

[4] Jefferies M (2018). Update on global epidemiology of viral hepatitis and preventive strategies. World J Clin cases.

[5] Cox AL (2020). Progress towards elimination goals for viral hepatitis. Nat Reviews Gastroenterol Hepatol.

[6] Malik GF (2022). Viral hepatitis-the road traveled and the journey remaining. Hepatic Medicine: Evidence And Research.

[7] Wu J et al. *Diagnosis, Treatment, and Prognosis of Viral Hepatitis*. Front Med, 2022. 9. 10.3389/fmed.2022.882878 PMC9096095 35572971

[8] Kong H (2021). Advanced nanotheranostics of CRISPR/Cas for viral hepatitis and hepatocellular carcinoma. Adv Sci.

[9] Barrangou R (2007). CRISPR provides acquired resistance against viruses in prokaryotes. Science.

[10] Ishino Y (1987). Nucleotide sequence of the iap gene, responsible for alkaline phosphatase isozyme conversion in Escherichia coli, and identification of the gene product. J Bacteriol.

[11] Makarova KS (2020). Evolutionary classification of CRISPR–Cas systems: a burst of class 2 and derived variants. Nat Rev Microbiol.

[12] O’Connell MR (2019). Molecular mechanisms of RNA targeting by Cas13-containing type VI CRISPR–Cas systems. J Mol Biol.

[13] Koonin EV, Makarova KS, Zhang F (2017). Diversity, classification and evolution of CRISPR-Cas systems. Curr Opin Microbiol.

[14] Makarova KS (2015). An updated evolutionary classification of CRISPR–Cas systems. Nat Rev Microbiol.

[15] Shmakov S (2015). Discovery and functional characterization of diverse class 2 CRISPR-Cas systems. Mol Cell.

[16] Shmakov S (2017). Diversity and evolution of class 2 CRISPR–Cas systems. Nat Rev Microbiol.

[17] Zuckerman JN, Zuckerman AJ, Heights M (2010). *Hepatitis viruses* infectious Diseases.

[18] Tu T, Patel K, Shackel N. Chap. 1*7-Viral Hepatitis* Genomic and, 2017.

[19] Jha V et al. *Computational Screening of Medicinal Plant Phytochemicals to Discover Potent Inhibitors against Hepatitis B Virus*

[20] Manns MP, Maasoumy B. Breakthroughs in hepatitis C research: from discovery to cure. Nature Reviews Gastroenterology & Hepatology; 2022. pp. 1–18. 10.1038/s41575-022-00608-8 PMC9122245 35595834

[21] Trujillo-Ochoa JL, Viera-Segura O, Fierro NA (2019). Challenges in management of hepatitis a virus epidemiological transition in Mexico. Ann Hepatol.

[22] Feld JJ. *Chronic viral hepatitis in adults and children: hepatitis B* Hepatology: Diagnosis and Clinical Management, 2012: p. 185.

[23] Sharapov UM. Hepatitis A, Foodborne Infections and Intoxications. 2013,Elsevier. 279–86.

[24] Castaneda D (2021). From hepatitis A to E: a critical review of viral hepatitis. World J Gastroenterol.

[25] Migueres M, Lhomme S, Izopet J (2021). Hepatitis A: epidemiology, high-risk groups, prevention and research on antiviral treatment. Viruses.

[26] Michaelis K (2018). Hepatitis A virus infections, immunisations and demographic determinants in children and adolescents, Germany. Sci Rep.

[27] Mikhailov MI (2020). Universal single-dose vaccination against hepatitis A in children in a region of high endemicity. Vaccines.

[28] Gupta S. *Viral Hepatitis: Historical Perspective, Etiology, Epidemiology, and Pathophysiology* Gupta, S.(primera edición), Studies on Hepatitis Viruses: Life Cycle, Structures, Functions, and Inhibition (págs. 1–14). Elsevier, 2018.

[29] Beck J, Nassal M (2007). Hepatitis B virus replication. World J gastroenterology: WJG.

[30] Logoida M (2021). Comparison of two diagnostic methods for the detection of Hepatitis B virus genotypes in the Slovak Republic. Pathogens.

[31] Kafeero HM (2022). Hepatitis B virus (HBV) serological patterns among the HBsAg negative hospital attendees screened for immunization. Sci Rep.

[32] Akbar SMF (2021). The Safety and Efficacy of a therapeutic vaccine for chronic Hepatitis B: a Follow-Up study of Phase III Clinical Trial. Vaccines.

[33] McQuillan GM (1999). Prevalence of hepatitis B virus infection in the United States: the National Health and Nutrition examination surveys, 1976 through 1994. Am J Public Health.

[34] Liang T, Hepatitis B. *The virus and disease. Hepatology, 49*. S13–S21. 10.1002/hep, 2009. 22881. 10.1002/hep.22881 PMC2809016 19399811

[35] Diogo Dias J, Sarica N, Neuveut C. *Early Steps of Hepatitis B Life Cycle: From Capsid Nuclear Import to cccDNA Formation*. Viruses, 2021. 13(5). 10.3390/v13050757 PMC8145197 33925977

[36] Xia Y, Guo H (2020). Hepatitis B virus cccDNA: formation, regulation and therapeutic potential. Antiviral Res.

[37] Edey M, Barraclough K, Johnson DW (2010). Review article: Hepatitis B and dialysis. Nephrol (Carlton).

[38] Smalls DJ (2019). Hepatitis B Virus Reactivation: risk factors and current management strategies. Pharmacotherapy: The Journal of Human Pharmacology and Drug Therapy.

[39] Syed G, Wyles D, Siddiqui A. Hepat Viruses. 2014.

[40] Ju W (2015). Hepatitis C virus genotype and subtype distribution in chinese chronic hepatitis C patients: nationwide spread of HCV genotypes 3 and 6. Virol J.

[41] Gupta P (2013). Hepatitis C virus and HIV type 1 co-infection. Infect disease Rep.

[42] Zheng Y et al. *Global Burden and Changing Trend of Hepatitis C Virus Infection in HIV-Positive and HIV-Negative MSM: A Systematic Review and Meta-Analysis*. Front Med, 2021. 8. 10.3389/fmed.2021.774793 PMC8710739 34966758

[43] Backmund M (2005). Hepatitis C virus infection and injection drug users: prevention, risk factors, and treatment. Clin Infect Dis.

[44] Kim CW, Chang K-M (2013). Hepatitis C virus: virology and life cycle. Clin Mol Hepatol.

[45] Colpitts CC, Tsai P-L, Zeisel MB. *Hepatitis C virus entry: An intriguingly complex and highly regulated process* International Journal of Molecular Sciences, 2020. 21(6): p. 2091. 10.3390/ijms21062091 PMC7140000 32197477

[46] Gupta E, Bajpai M, Choudhary A (2014). Hepatitis C virus: screening, diagnosis, and interpretation of laboratory assays. Asian J Transfus Sci.

[47] Mu J-J (2004). The small delta antigen of hepatitis delta virus is an acetylated protein and acetylation of lysine 72 may influence its cellular localization and viral RNA synthesis. Virology.

[48] Sausen DG, et al. Hepatitis B and Hepatitis D Viruses: a Comprehensive Update with an immunological focus. Int J Mol Sci. 2022;23. 10.3390/ijms232415973. 10.3390/ijms232415973 PMC9781095 36555623

[49] USTIANOWSKI A, K. HANDBOOK OF SYSTEMIC, AUTOIMMUNE, DISEASES. 2020. 16: p. 59–82.

[50] Olivero A, Smedile A. Hepatitis delta virus diagnosis. Seminars in liver disease. 2012.Thieme Medical Publishers. 10.1055/s-0032-1323627 22932970

[51] Kim J-H (2014). A systematic review of the epidemiology of hepatitis E virus in Africa. BMC Infect Dis.

[52] Shieh Y, Cromeans T, Sobsey M. *VIRUSES| hepatitis viruses transmitted by food, water, and environment* 2014.

[53] Chevaliez S, Pawlotsky J-M. Hepatitis Viruses, Infectious Diseases. 2017,Elsevier. 1417–25.

[54] Nelson KE, Labrique AB, Kmush BL. Epidemiology of genotype 1 and 2 hepatitis E virus infections. Volume 9. Cold Spring Harbor perspectives in medicine; 2019. p. a031732. 6. 10.1101/cshperspect.a031732 PMC6546036 29735579

[55] Nicot F (2021). Classification of the zoonotic hepatitis E virus genotype 3 into distinct subgenotypes. Front Microbiol.

[56] Hartl J, Wehmeyer MH, Pischke S (2016). Acute hepatitis E: two sides of the same coin. Viruses.

[57] Zhao C, Wang Y. *Laboratory diagnosis of HEV infection*. Hepat E Virus, 2016: p. 191–209. 10.1007/978-94-024-0942-0_11 27738986

[58] Talapko J et al. Towards the improved accuracy of hepatitis e diagnosis in vulnerable and target groups: A global perspective on the current state of knowledge and the implications for practice. Healthcare. 2021.MDPI. 10.3390/healthcare9020133 PMC7912707 33572764

[59] Sime-Ngando T (2014). Environmental bacteriophages: viruses of microbes in aquatic ecosystems. Front Microbiol.

[60] CJ B, CR H. and M. FA, - laboratory diagnosis of Virus Diseases. Fenner and White’s Medical Virology; 2017.

[61] Xiao M (2022). Virus detection: from state-of-the-Art Laboratories to Smartphone-Based point-of-care testing. Adv Sci.

[62] Dronina J, Samukaite-Bubniene U, Ramanavicius A (2021). Advances and insights in the diagnosis of viral infections. J Nanobiotechnol.

[63] Yuan B, et al. Application of the CRISPR/Cas System in Pathogen detection: a review. Molecules. 2022;27. 10.3390/molecules27206999. 10.3390/molecules27206999 PMC9610700 36296588

[64] Chertow DS (2018). Next-generation diagnostics with CRISPR. Science.

[65] Hille F (2018). The Biology of CRISPR-Cas: Backward and Forward. Cell.

[66] Kaminski MM (2021). CRISPR-based diagnostics. Nat Biomedical Eng.

[67] Bao W, Jurka J (2013). Homologues of bacterial TnpB_IS605 are widespread in diverse eukaryotic transposable elements. Mob DNA.

[68] Chen JS (2018). CRISPR-Cas12a target binding unleashes indiscriminate single-stranded DNase activity. Science.

[69] Zetsche B (2015). Cpf1 is a single RNA-Guided endonuclease of a class 2 CRISPR-Cas System. Cell.

[70] Ding R (2021). CRISPR/Cas system: a potential technology for the Prevention and Control of COVID-19 and Emerging Infectious Diseases. Front Cell Infect Microbiol.

[71] Xiang X (2020). CRISPR-cas systems based molecular diagnostic tool for infectious diseases and emerging 2019 novel coronavirus (COVID-19) pneumonia. J Drug Target.

[72] Chen K (2022). Research progress of CRISPR-based biosensors and bioassays for molecular diagnosis. Front Bioeng Biotechnol.

[73] Puig-Serra P, et al. CRISPR approaches for the diagnosis of Human Diseases. Int J Mol Sci. 2022;23. 10.3390/ijms23031757. 10.3390/ijms23031757 PMC8836363 35163678

[74] Chakraborty J (2022). CRISPR/Cas-Based Biosensor as a New Age Detection Method for pathogenic Bacteria. ACS Omega.

[75] Lee RA et al. *Ultrasensitive CRISPR-based diagnostic for field-applicable detection of Plasmodium species in symptomatic and asymptomatic malaria* Proceedings of the National Academy of Sciences, 2020. 117(41): p. 25722–25731. 10.1073/pnas.2010196117 PMC7568265 32958655

[76] Kaminski MM (2020). A CRISPR-based assay for the detection of opportunistic infections post-transplantation and for the monitoring of transplant rejection. Nat Biomed Eng.

[77] Bruch R (2019). CRISPR/Cas13a-Powered Electrochemical Microfluidic Biosensor for Nucleic Acid amplification-free miRNA Diagnostics. Adv Mater.

[78] Shan Y (2019). High-fidelity and Rapid quantification of miRNA combining crRNA programmability and CRISPR/Cas13a trans-cleavage activity. Anal Chem.

[79] Gootenberg JS (2018). Multiplexed and portable nucleic acid detection platform with Cas13, Cas12a, and Csm6. Science.

[80] Hajian R (2019). Detection of unamplified target genes via CRISPR–Cas9 immobilized on a graphene field-effect transistor. Nat Biomedical Eng.

[81] Jolany vangah S (2020). CRISPR-Based diagnosis of Infectious and Noninfectious Diseases. Biol Procedures Online.

[82] Pardee K (2016). Rapid, low-cost detection of Zika Virus using Programmable Biomolecular Components. Cell.

[83] Kellner MJ (2019). SHERLOCK: nucleic acid detection with CRISPR nucleases. Nat Protoc.

[84] Wang S (2021). Rapid nucleic acid detection of Escherichia coli O157:H7 based on CRISPR/Cas12a system. Food Control.

[85] Mustafa Mujahed I, Makhawi Abdelrafie M (2021). SHERLOCK and DETECTR: CRISPR-Cas Systems as potential Rapid Diagnostic Tools for Emerging Infectious Diseases. J Clin Microbiol.

[86] Myhrvold C (2018). Field-deployable viral diagnostics using CRISPR-Cas13. Science.

[87] Li SY (2018). CRISPR-Cas12a-assisted nucleic acid detection. Cell Discov.

[88] Kumaran A, et al. Advancements in CRISPR-Based Biosensing for Next-Gen Point of Care Diagnostic Application. Biosensors. 2023;13. 10.3390/bios13020202. 10.3390/bios13020202 PMC9953454 36831968

[89] Li Y (2019). CRISPR/Cas Systems towards Next-Generation Biosensing. Trends Biotechnol.

[90] Aman R, Mahas A, Mahfouz M (2020). Nucleic acid detection using CRISPR/Cas Biosensing Technologies. ACS Synth Biol.

[91] Yin L (2021). CRISPR-Cas based virus detection: recent advances and perspectives. Biosens Bioelectron.

[92] Chen X (2021). A CRISPR-Cas12b-Based platform for Ultrasensitive, Rapid, and highly specific detection of Hepatitis B virus genotypes B and C in clinical application. Front Bioeng Biotechnol.

[93] Zhang X (2022). CRISPR/Cas13-assisted hepatitis B virus covalently closed circular DNA detection. Hepatol Int.

[94] Wang S (2021). Highly sensitive and specific detection of hepatitis B virus DNA and drug resistance mutations utilizing the PCR-based CRISPR-Cas13a system. Clin Microbiol Infect.

[95] Ding R et al. *CRISPR/Cas12-Based Ultra-Sensitive and Specific Point-of-Care Detection of HBV*. Int J Mol Sci, 2021. 22(9). 10.3390/ijms22094842 PMC8125043 34063629

[96] Ashraf MU (2021). CRISPR-Cas13a mediated targeting of hepatitis C virus internal-ribosomal entry site (IRES) as an effective antiviral strategy. Biomed Pharmacother.

[97] Wang H (2022). Rapid Visual detection of Hepatitis C Virus using reverse transcription recombinase-aided amplification-lateral Flow Dipstick. Front Cell Infect Microbiol.

[98] Kham-Kjing N et al. *Highly Specific and Rapid Detection of Hepatitis C Virus Using RT-LAMP-Coupled CRISPR-Cas12 Assay*. Diagnostics (Basel), 2022. 12(7). 10.3390/diagnostics12071524 PMC9317538 35885430

[99] Li H (2022). Amplification-free detection of SARS-CoV-2 and respiratory Syncytial Virus using CRISPR Cas13a and Graphene Field-Effect Transistors. Angew Chem Int Ed Engl.

[100] Huang Z, et al. Fluorescence Signal-Readout of CRISPR/Cas Biosensors for nucleic acid detection. Biosensors. 2022;12. 10.3390/bios12100779. 10.3390/bios12100779 PMC9599699 36290917

[101] Li P (2021). Applications of the CRISPR-Cas system for infectious disease diagnostics. Expert Rev Mol Diagn.

[102] Palaz F (2021). CRISPR-based tools: alternative methods for the diagnosis of COVID-19. Clin Biochem.

[103] Wang X, Shang X, Huang X (2020). Next-generation pathogen diagnosis with CRISPR/Cas-based detection methods. Emerg Microbes Infect.

[104] Ibrahim AU (2022). Futuristic CRISPR-based biosensing in the cloud and internet of things era: an overview. Multimed Tools Appl.

